# Sustained effects of an early childhood language and literacy intervention through second grade: Longitudinal findings of the SPELL trial in Denmark

**DOI:** 10.1371/journal.pone.0258287

**Published:** 2021-10-11

**Authors:** Dorthe Bleses, Philip S. Dale, Laura Justice, Anders Højen, Benedicte D. Vind, Hui Jiang

**Affiliations:** 1 School of Communication and Culture, Aarhus University, Aarhus C, Denmark; 2 TrygFonden’s Centre for Child Research, Aarhus University, Aarhus C, Denmark; 3 Department of Economics and Business Economics, Aarhus University, Aarhus C, Denmark; 4 Centre for Integrated Register-Based Research, CIRRAU, Aarhus University, Aarhus C, Denmark; 5 Department of Speech & Hearing Sciences, University of New Mexico, Albuquerque, NM, United States of America; 6 Crane Center of Early Childhood Research and Policy, Ohio State University, Columbus, OH, United States of America; University of Florida, UNITED STATES

## Abstract

Predictive relations between language and literacy skills during the preschool years and children’s future reading achievement are well-documented, leading to development and evaluation of preschool interventions targeting early skill development. Although educational researchers have developed and found some positive short- and mid-term effects of language and literacy intervention supplements implemented in early childhood education (ECE) settings, fade-out is a concern. Most studies have targeted children experiencing risk, rather than a more representative sample. Additionally, there are very few studies of long-term intervention effects, and heterogeneity of long-term effects has not been well described. In the present study, we build on initial reports of one of the largest studies of a language and literacy intervention supplement, the SPELL randomized controlled trial implemented as part of the universal ECE system in Denmark. SPELL was delivered to an unselected sample of children at 3–5 years of age (*n =* 7,076). Results of the base intervention (SPELL) and two enhanced versions featuring extended professional development for teachers (SPELL+PD) or an add-on home-based program for parents (SPELL+HOME) showed short-term effects for literacy outcomes for all children for all SPELL conditions compared to business as usual (BAU). In this follow-up study, we utilized follow-up assessments of 2,700 SPELL 4-5-year-old participants with national reading tests in second grade. The main analyses based on the whole sample showed no significant differences in reading scores in second grade for those in any of the three SPELL conditions relative to the BAU condition. However, moderation analyses demonstrated heterogeneity in intervention effects with children whose mothers had low-mid education showing sustained and mostly large-sized effects. Other risk factors, including income and immigrant background, and condition interacted with at least one outcome variables. These findings suggest that at-risk children in some cases derive long-term benefits from early language and literacy intervention enhancing learning opportunities in ECE settings.

## Introduction

For more than two decades, educational researchers have developed and evaluated for efficacy a specific set of practices, hereafter ‘intervention supplements’, designed to enhance the early language and literacy skills of preschool-aged children [1–6]. The premise for this work resides in the well-documented predictive relations between language and literacy skills during the preschool years and children’s future reading achievement in both word recognition and reading comprehension [7–9]. Language skills early in life are among the strongest predictors of language skills and academic performance in school; a recent prospective community cohort study of 1,910 infants found that whereas early life factors (e.g., birth-related factors, maternal education, family history, and mental health) only explained up to 14% of variance in academic scores at 11 years, including language scores from 2 to 11 years increased the amount of variance explained up to 54% [10]. In fact, poor skills in several domains of language and literacy early in life have turned out to be a distinct risk factor for later language and reading outcomes [11].

A large body of research has documented sources of variation in children’s early language and literacy development beyond the factors mentioned above. Substantial variation in children’s early language development has, for instance, been linked to parental demographic characteristics such as socio-economic status (SES, especially as indexed by parental education) and immigrant status (especially language minority homes). Children from low-SES families and/or language minority homes have increased risk of reaching school age with lower language levels compared to children from monolingual families with mid- and higher SES [12]. Even in the welfare state of Denmark, with its universal early childhood education (ECE) system, immigrant background is a strong negative predictor of language and literacy skills in the preschool years. A recent study explored how parental SES and region of origin for four groups of immigrants interacted in 12,470 children from 2 to 6 years of age and found immigrant background consistently predicted lower language skills [13].

### Short-term effects of language and literacy intervention supplements

These research conclusions, even though primarily based on correlational evidence, have provided plausibility to the hypothesis that enhancing the language and literacy skills of young children, particularly those who show lags in skill development, may lead to short- and mid-run improvements in language and consequently later reading achievement, as later attainments build on foundations that are laid down earlier [14]. Long-term benefits might result directly from the cumulative nature of reading development as well as indirectly from child effects on parents and teachers [15, 16]. There is considerable evidence that intervention supplements implemented within ECE settings can significantly improve early language and literacy skills by improving the quality of the learning environment in the ECE setting. Such intervention supplements are important due to varying quality of the learning environment in ECE settings [17]. Several meta-analyses have synthesized this body of work to guide future research efforts and inform intervention practices in ECE settings [e.g., 18–20].

The majority of such language and literacy interventions implemented in ECE settings have included interactive book reading as a central intervention component. For instance, Bierman and colleagues showed that exposure to a multi-component intervention (including interactive book reading) focused on language, literacy, and social-emotional skills during preschool had positive short-term effects on a range of cognitive and social-emotional skills including early vocabulary development (mean effect size 0.15) and emergent literacy skills (mean effect sizes between 0.16–0.39) [21]. Similarly, Justice and colleagues developed and tested an intervention, Project STAR (Sit Together and Read), a 30‐week program during which preschool teachers systematically referenced print during shared reading, which improved print knowledge over the school year (mean effect size 0.21) for the children in the treatment group [22]. Finally, Whitehurst et al. [23] implemented a program which combined small group dialogic reading sessions with a phonological intervention component (the Sound Foundations program) in Head Start preschools over a school year. Posttest outcomes resulted in significant effects for print concepts (mean effect size 0.13) but not for a measure of phonological awareness (mean effect size 0.10) or language (mean effect size 0.10).

As shown by these results, the magnitude of intervention effect is highly variable, and in particular in studies with larger samples, which produce more precise effect sizes, often is less than 0.3 [24]. This is relevant because most often, effect sizes are interpreted on the basis of benchmarks proposed some time ago by Cohen [25]: 0.2 = small, 0.5 = medium, 0.8 = large. Consequently, effect sizes of intervention research, which often are smaller than Cohen’s 0.2 benchmark in size, are viewed as small or less. However, Kraft and others [26] recently argued that these benchmarks are inappropriate due in part to the nature of intervention research methodology. Consequently, Kraft proposed benchmarks to interpret effects of RCTs of educational interventions with more appropriate measures as less than 0.04 = small, 0.05 to less than 0.20 = medium, and 0.20 or greater = large. Based on these benchmarks, many interventions which have focused on early-learning enhancements would be classified as medium-sized. Kraft also argued that cost and scalability of interventions merit equal consideration in evaluation. For example, providing breakfast has a medium effect size at low cost and high scalability, whereas school facility improvements produce a medium effect at high cost, but also high scalability.

### Longer-term effects and non-effects of ECE intervention supplements

As evidence has accumulated that early intervention supplements can facilitate early language and literacy skills in children, the issue of partial or even complete “fade-out of gains of early interventions have become increasingly prominent. Returning to the Bierman et al. study, for example, a follow-up study in kindergarten one year after the original study demonstrated that the early intervention led to heightened decoding skills (mean effect size 0.15) in kindergarten for children who participated in the Head start REDI program. There was no significant improvement for vocabulary (mean effect size 0.10), letter-word identification (mean effect size 0.03) or sight word efficiency (mean effect size -0.04) [27]. Subsequent follow-up in Grade 5 showed sustained effects on a measure of academic engagement (mean effect size 0.28), but no measures of actual academic skills were evaluated [28]. Whitehurst et al. conducted a follow-up assessment of the combined dialogical reading and phonological awareness program in second grade, and did not find any significant effects on measures of word reading and word attack [23].

More broadly, there is considerable evidence that the benefits of such interventions often do not last throughout elementary school and, in fact, fade out by the end of kindergarten or first grade [29]. Although the term fadeout is commonly used to describe this phenomenon, in most cases, it is children who did not receive the preschool intervention that catch up to the skill level of the children who did. Nevertheless, understanding why these patterns of skill development occur is key to maximizing the effect of investing in early education. In their conceptual piece, Bailey and colleagues introduced the concepts of skill building models, “foot in the door” interventions and sustaining environments [30]. Here we focus on skill building models and sustaining environments that are most relevant for the aims of the current paper.

Key to skills building models is that simpler skills lay the foundation for the later development of more advanced skills. Bailey et al. introduce the concept of “Trifecta skills” which are those skills that are malleable, fundamental, and would not have developed in the absence of the intervention. These skills increase the productivity of later investments and predict greater impact persistence of early human capital interventions. Early language and literacy skills, supported in early language and literacy interventions, are an example of skills that are malleable and lay the foundation for later reading. However, according to Bailey et al., they do not possess the third trifecta condition, because most children are likely to acquire these skills soon after entering school, at least at some level. This is particularly true for the more constrained skills (e.g., letter knowledge), in contrast to non-constrained skills like vocabulary which can always be expanded. On the other hand, Bailey et al. acknowledge that a skill might qualify as in the trifecta in certain kinds of adverse environment.

The concept of sustaining environments focuses on the importance of environments experienced after an intervention concludes [30]. The elementary classroom is a key environment experience that has the potential to prevent fadeout of preschool interventions. Children who experience high quality interventions in preschool may have lower quality classroom experiences than needed in kindergarten, which disrupts their learning and may contribute to apparent “catch-up” by control children. For example, Ansari and Pianta [31] found that when children who attended high quality early childcare programs also experienced high quality elementary school environments, the benefits from their early childcare programs lasted through age 15. This suggests that improving elementary school classroom quality may maintain or even amplify the gains produced through early childhood investments. Another, related explanation for the lack of sustained effects of early interventions is that preschool and elementary school classrooms are not aligned neither in academic content or instructional methods, for instance use of direct instruction, role play, craft, technology and free play [32, 33]. As a consequence, children experience large differences in their day-to-day experiences that disrupt, and potentially negate, the learning advantages they gained in high quality preschool. Although it is clear that instructional methods are distinct between preschool and elementary school, there is also a concern that the academic content of what is being taught is overlapping and that failing to build on preschool learning will reduce the continuing benefit of preschool learning. For example, recent national data in the U.S. revealed that 37% of the language, literacy, and math content covered in kindergarten is redundant with content covered in preschool [34]. Other work has shown that focusing on basic, as opposed to advanced, academic skills in kindergarten is associated with lower learning [35]. Taken as a whole, this recent body of work suggests that without improving the classroom quality children experience in early elementary school by aligning with, and building on, the academic content and instructional methods used in preschool, the full potential benefits of preschool intervention supplements may not be realized.

Finally, an additional factor mentioned by Bailey et al. [36], in a comprehensive review of factors underlying fade-out or persistence of early interventions, is over-alignment between treatments and outcomes, or, on a larger scale, the problem of multidimensionality. Early education may successfully target specific aspects of the problem, but even if that specific effect is maintained, successful later performance may require a wider range of skills, most of them not addressed earlier. Transfer of learning appears to be more difficult, and hence more modest in scope, than has often been assumed.

### Heterogeneity of intervention effects for sub-populations

A second emerging issue for ECE intervention supplements is the need to move beyond average effects to determine if there are systematic differences reflecting identifiable subgroups. In some cases, this has led to more detailed analyses of the participants in the study, whereas in others it has motivated broadening the target sample. For example, the largest proportion of the studies testing language and literacy intervention supplements in ECE settings in the U. S., at least, has focused on programs and enhancement interventions serving children experiencing demographic or biomedical risk [3, 21, 37, 38], reflecting social values of opportunity and equality. It is often the goal of intervention to benefit primarily the children who are at greatest risk, or already showing the poorest performance. The importance and challenge of this goal are even greater because of the demonstrated existence of ‘Matthew Effects’, whereby the children who gain the most from educational experience are those who are already doing better than the average [39]. Reversing this direction of heterogeneity is a social value.

The study by Wasik and colleagues [3] is an example of an intervention that targeted only children experiencing social risk. The authors developed and tested a vocabulary-focused intervention in Head Start classrooms, a federally funded program that provides preschool programming to children from low-income households. Their work showed that children’s vocabulary growth could be accelerated through structured book-reading activities. Such work is important given extensive evidence showing that children from low-income homes and/or who exhibit, on average, developmental disabilities experience significant lags in their development of language and literacy skills, including those that are important precursors to skilled reading [12, 40, 41].

However, universal ECE programs including both at-risk and non-at-risk populations are emerging in the U.S. and are quite common in Europe. This is, for instance, the case in Denmark which has implemented a universal and highly subsidized ECE system from infancy to school start; three out of four children are enrolled in childcare before they turn 3 years and 90% of all children spend 5 years in total in childcare [42]. Yet, recent work has suggested that the quality of universal ECE programs in terms of providing children with adequate learning opportunities is low. An international comparison has suggested that the quality of ECE settings in Denmark may not be sufficient to promote learning and learning in children [43]. Furthermore, evidence from Denmark specifically reveals large achievement gaps between at-risk and non-at-risk children in preschool [13], schools [44] and in final educational attainment [45]. At the other end of the scale, studies indicate that Denmark also has a lower percentage of high performing readers than in other comparable countries like Sweden and Finland [46]. Consequently, it is important to understand whether intervention supplements in universal ECE systems support the short- and long-term language and literacy development in all children or whether their effects are limited to children exhibiting risk, as documented in, for instance, work in the U.S [24]. Different risk indicators may also lead to differential effectiveness of intervention. As Dale et al. [47] pointed out, low-SES subgroups and clinically low-scoring subgroups appear to respond differently to language interventions.

#### Low-cost language and literacy interventions tested at-scale

One of the largest randomized controlled trials (RCT) of a language and literacy intervention supplement conducted thus far was the SPELL project, implemented in the universal ECE setting in Denmark, and therefore delivered to an unselected sample of children [48]. The main aim of the intervention was to increase the language and literacy learning opportunities in ECE settings for all children with the overall aim of investigating if and for whom such an intervention supplement would improve language and literacy skills.

The core of the SPELL study was the development and implementation of an adaptation of Read It Again-PreK, a whole-class shared book reading intervention involving two lessons weekly that target four domains of language and literacy skill, namely vocabulary, narrative, print knowledge and phonological awareness [1]. The tool is freely available, can be adapted with permission (see *Acknowledgement*), and was designed to be very low-cost, largely requiring only a set of commercial-grade storybooks and typical classroom materials. A Danish team of researchers developed and piloted the adapted intervention, titled Structured Preschool Efforts in Language and Literacy, and subsequently conducted a large-scale country-wide effectiveness study of its impacts on an unselected sample of children (*n =* 7,076). Read It Again-PreK was adapted to the Danish context and language with two main changes: the overall structure of the intervention, involving twice weekly whole-class sessions for 30 weeks, was redesigned to 20 weeks, and the format changed from whole-class to small-group intervention to better fit the Danish ECE context. Explicit costs (including paying for substitutes while the staff participated in the two-day professional development course and expenses to intervention materials) were calculated for the three SPELL conditions, and the total individual student cost for implementing the intervention was 61 USD for SPELL, 77 USD for SPELL+PD and 146 USD for SPELL+Home [49].

The trial evaluated teachers’ implementation of SPELL in their classrooms for a 20-week period, comprising two lessons delivered weekly to children in small groups of about 4–6 children, as well as two additional planned variations: SPELL+PD, in which teachers received additional professional development (14 hours, targeting quality implementation of SPELL components and use of individual children’s language development profiles), and SPELL+HOME, in which children’s caregivers implemented two SPELL-aligned activities at home over the intervention period. Results showed that exposure to all three intervention conditions (SPELL, SPELL+PD, SPELL+HOME) significantly improved children’s literacy skills based on a composite of three tasks (*ds* for the three conditions were 0.22, 0.27, and 0.21, respectively; i.e., large effects in the Kraft rubric) but had no significant effect on children’s language skills (*ds* 0.16, 0.13, and 0.04, respectively).

The large and diverse sample enabled moderation analyses for subgroups of particular interest, namely mother’s education and immigrant status (i.e., either the child or both parents were immigrants). These subgroups are of particular interest in a Danish context because substantial and significant skill gaps associated with low SES (indexed by household income and immigrant status) have been documented in Denmark [13]. Consistent with this pattern, in a separate analysis of pretest data from the SPELL study, higher maternal education predicted higher language skills whereas immigrant status predicted lower language skills [48]. Specifically, there was a difference of approximately one standard deviation on vocabulary development between children whose mothers had no post-secondary education vs. those with higher education. Similarly, in the same dataset there were even larger gaps between children with immigrant status (the majority of whom are learning Danish as a second language), and non-immigrant children. These findings are perhaps surprising as Denmark, as described above, is a welfare state with an ECE system featuring universal enrollment of children [42]. Returning to the effects of SPELL, moderational analyses based on the often-examined moderators, gender and age, as well as moderation analyses based on mother’s education and immigrant status did not, however, show any differential effects [48]. An additional in-depth analysis of the SPELL results did show that children with low initial skills (as opposed to children with low maternal education, a risk condition) benefited significantly more from SPELL exposure than other children [47]. Nevertheless, these results indicate that a low-cost intervention supplement implemented in the universal ECE setting in Denmark also improved learning opportunities for the highest performing children as well.

### The present study

The large-scale SPELL RCT study conducted in the Danish universal ECE context offers a rare opportunity to consider long-term effects of a language and literacy intervention supplement on the future achievement of school-aged children. As a relatively small welfare country, Denmark has a centralized data repository that carefully tracks data on children’s education performance from second to eighth grade, and The Danish Ministry of Children and Education has made the national Danish test data available for researchers. Thus, it is possible to consider long-term impacts for those children in the SPELL sample who took the national reading test in second grade.

A longitudinal study of long-term effects of SPELL on academic outcomes can add to the research base in several ways. First, whereas most long-term studies of early language and literacy intervention supplements in fact assess mid-term effects (one or two years after the intervention), the long-term effects of SPELL were assessed 3 to 6 years post intervention. Second, the short-term outcomes were evaluated on broader measures of language and literacy skills, avoiding overalignment between treatment and outcomes. Third, the original SPELL study was based on a large-scale study implemented in a universal ECE context with a relatively population-representative sample of several thousand children. There have been very few studies of longitudinal intervention effects from language and literacy intervention supplements implemented in universal ECE settings [24]. It is therefore unknown whether language and literacy intervention supplements implemented in universal ECE programs exert longer-term advantages to all or perhaps one or more subgroups of children. Due to the large size and heterogeneous nature of the sample, it is possible to investigate the heterogeneity of intervention effects for both at-risk and non-at-risk children (as identified by sociodemographic variables and child skills), which can also throw light on effects of quality investments in universal ECE settings.

The present study addressed two primary research aims. The first aim was to examine the extent to which the SPELL intervention influenced average reading achievement in second grade, in particular children’s language comprehension, word decoding, and reading comprehension, or if partial or complete fade-out occurred. The second aim was to explore heterogeneity in long-term impacts focusing on factors at child level (gender, age, immigrant status, and initial skills) and parent level (maternal education and income) known to be associated with child development. Although these factors, other than initial skills, were not found to predict effectiveness in the short-term analyses, they are contextual factors which continue through childhood and may have different long-term importance for at-risk vs. non-at-risk children.

## Methods

### Participants and procedure

Data for the original SPELL RCT were collected between November 2013 and June 2014 in two consecutive cohorts of implementation which included a total of 144 childcare centers serving children 3 to 6 years of age across Denmark. Random assignment to one of four conditions comprising three SPELL treatments (SPELL, SPELL+PD, SPELL+HOME) or business-as-usual (BAU) was conducted at the level of the center, resulting in 36–38 centers per condition and a five-level study design (center, classroom, teacher, group, child), as detailed in the original paper [48]. As randomized, the study involved 561 teachers and 7,076 children although there was some attrition over time for both teachers and children. The number of childcare centers per condition varied marginally at pretest (BAU, *n =* 36; SPELL, *n =* 38; SPELL+PD, *n =* 34; SPELL+HOME, *n =* 34).

For the present purposes, the sample was constrained to only include children who were 4- or 5-year-olds during the original SPELL study, firstly because the assessment battery of the 3-year-olds differed from that of the 4-6-year-olds and secondly because a smaller proportion of the 3-year-old children in the original sample had reached second grade when the present analyses were conducted. In addition, we excluded children who were 6 years old in the original study, as there were relatively few children in this group (*n =* 123 of 2823) and because in the Danish context this group of children typically has been retained in preschool for a longer duration than other children due to developmental or behavioral problems. Finally, children in the three SPELL treatment conditions were excluded if fidelity data showed that they did not receive the intervention. Specifically, during study implementation, data on children’s attendance in SPELL sessions was captured by teachers, and there was a subset of children who were identified as having never participated in any SPELL sessions (*n =* 14 of 2714). These children were excluded in the present analyses, as our goal was to estimate long-term effects for children who were exposed to SPELL during their preschool years.

Children’s reading skills were assessed through the Danish mandatory national reading test administered to all students in second grade (see below) in the spring of 2016, 2017, 2018, or 2019, depending on the children’s age at the time of the SPELL intervention. Consequently, the reading test took place 3 to 6 years after children received the SPELL intervention. The Danish national reading test is implemented by The Danish Ministry of Children and Education and the data were merged with our sample using the Danish Central Personal Number System. In total, we were able to match SPELL participants with reading tests in second grade for 2,700 4-5-year-old children, representing 81% of the originally randomized sample for these age groups. The second grade data included scores from 701 children in the BAU condition, 770 in SPELL, 692 in SPELL+PD and 537 in SPELL+HOME treatment group.

The Danish Data Protection Agency approved the collection and treatment of all data for the project. Due to the registration with the Danish Data Protection Agency, the project is categorized as public research. For public research projects of significant societal importance, it is not required to ask for consent from each parent in Denmark. Participants’ right to privacy, confidentiality and anonymity was strictly observed. The study adhered to relevant provisions outlined in The Act on Processing of Personal Data.

### Measures

#### Language and literacy skills during intervention

In the original RCT study, children’s language and literacy skills were assessed pre- and post-intervention by their teacher using a published assessment instrument, Language Assessment of Children [50, 51]. The tests represent skills targeted in the SPELL intervention (e.g., phonological awareness, print knowledge). The four subtests included measures of deletion (maximum score 20), which represents the child’s ability to segment sounds (*α =* .91); letter identification (maximum score 12), which represents the child’s knowledge of the alphabet letters and names (*α =* .86); vocabulary (expressive, maximum score 76), representing the child’s breadth of expressive words (*α =* .97); and comprehension (maximum score 27), representing the child’s understanding of words and complex sentences (*α =* .80). Criterion validity [48] is supported by significant, positive correlations (*r* = .42 to .57) with subscales from the Danish version of the Peabody Picture Vocabulary Test [52] and the Danish Expressive Vocabulary Test [53].

#### Reading skills at follow-up

The reading test in second grade is standardized and mandatory in all public Danish schools and in an IRT-based computerized adaptive system. The Danish National Tests are administered at the end of the school year. The system ensures that children’s results are comparable across municipalities, schools and classrooms, since the system evaluates children by the same standards and teachers are not involved in test administration or scoring. Each student is presented with a sequence of items of increasing difficulty levels based on performance on previous items, where the difficulty level of the items and student ability is measured on the same logit scale. The items are multiple-choice questions with a varying number of options. The Danish National Test in reading evaluates students’ ability within three areas: language comprehension (semantics of individual words, homonyms, language use, and idioms), decoding (word identification in concatenated words and word reading), and reading comprehension (comprehension of written texts). All tasks within the three areas require some text reading, but students with language problems can use text-to-speech facilities. Due to the individual adaptiveness of the test, it is not possible to calculate internal reliability measures. The correlations between the three reading tests range from *r =* .54 to .75. The Danish national tests have been shown to provide a valid estimate of student abilities based on predictive analyses; specifically, when national test scores were regressed on ninth grade school examinations for the same-subject national test data, the earlier national test results explained about 49% of the Danish examination marks, thus providing an external measure of validity [54].

#### Covariates

Information about age and gender was collected in the original study. Background information with respect to mother’s and father’s education, family income, and the child’s immigrant status (i.e., whether the child had no immigrant background as opposed to the child or both parents being immigrants) was obtained from Statistics Denmark, using the Danish Central Personal Number System. These measures served as covariates in this study. The information was obtained for the most recent year available at Statistics Denmark (parent’s education: 2017, family income: 2015, the child’s immigrant status: 2018). Following the categorization used by Statistics Denmark, mother’s and father’s education were divided into four groups: low (no education or elementary school as highest level), low-mid (vocational education or upper secondary school as highest level), mid-high (some post-secondary education or specialized training, such as teacher or nurse), and high (4-year post-secondary education or more as highest level).

### Analytic strategy

Given the longitudinal and education-based nature of our data, participating children were doubly nested, first in classrooms within childcare centers, where the SPELL interventions took place, and later within their elementary schools when the national tests were conducted. Intra-class correlations (ICCs) provided evidence for variance related to the random clustering effects. For classrooms in childcare centers, ICCs ranged from .14 for both language comprehension and reading comprehension to .16 for decoding; for the second grade classrooms within elementary schools, ICCs ranged from .13 for decoding to .18 for language comprehension; and for the crossing between second grade classroom and childcare classroom, the ICCs ranged from .07 for language comprehension to .11 for reading comprehension. Finally, at the child level, ICCs range from .57 for reading comprehension to .62 for decoding. These values indicate that the majority of variance was between children, but each random clustering effect contributed some true variation. In order to take the cross-classified nature of the longitudinal data into account in addressing the first research question, we used mixed-effects models that included the random clustering effects for both childcare classrooms and second grade classrooms. To account for any potential interaction effects between childcare centers and elementary schools, we further added the crossing between second grade classroom and childcare classroom as an additional random effect. We estimated three separate mixed-effects models, one for each outcome variable from the second grade test (language comprehension, decoding and reading comprehension). We included the following covariates at the child level: the child’s immigrant status, age, gender, and pretest scores from the language and literacy assessment that took place at the start of the SPELL intervention (scores in deletion, letter knowledge, vocabulary and comprehension). We also included the logarithm of family income as a covariate. As Statistics Denmark has data on parent education only for those who completed their schooling in the country, information on education for immigrant parents is either lacking or is not sufficiently comparable to that of native Danish children. As this would introduce potential bias in the analysis, we did not control for parents’ educational attainment, following the original SPELL paper [48]. All three outcome measures at second grade were standardized to have a mean value 0 and standard deviation 1 based on the results of the national reading test in second grade in the same year for all children in Denmark.

To answer our second research question, concerning moderation of the intervention effects, mixed effects models with all relevant interaction terms were initially estimated and nonsignificant interactions were dropped; the model was estimated again until only significant interactions remained. For interactions with gender, girl was the reference group, and the coefficients indicate the change of estimate when going from girl to boy. For interactions with education level, low was the reference group, and the coefficients indicate the change of estimate when going from low to each of the other three education levels. For interactions with immigrant status, native was the reference group and the coefficients indicate the change of estimate when going from native to immigrant. For the moderation analysis of education, we only included ethnic Danish children for the reasons mentioned above, and accordingly, immigrant background was dropped as a covariate. Pretest scores and the logarithm of income were continuous measures in the moderation model.

Missing data were handled with multiple imputation. All control variables (see above) but education were imputed using multiple imputation by chained equations [55] creating five imputed datasets. Robustness analyses creating 20 datasets did not change the results. As the information about immigrant parents’ education was lacking or was not sufficiently accurate, we did not use information about education when imputing data.

All analyses were done in STATA 15. The mixed-effects models were estimated using the STATA mixed command.

## Results

### Preliminary findings

Preliminary analyses were conducted to examine the extent to which the children with national tests differed from those who did not take the national test in the second grade.

Not all children in the original SPELL RCT completed the national reading test in second grade, and therefore the second grade test scores were not available for them. There are no official statistics describing why some children do not take the national tests, but among reasons often mentioned are school absence on the specific test day (due to sickness or parents deliberately keeping their child at home due to discomfort with testing of children), or children not being given the test because they are judged unable (by either teacher or parent) to take it, mainly because of poor language skills or social-emotional problems. In Table 1, we provide a comparison of the longitudinal sample for whom the second grade test were (*n* = 2700) or were not (*n* = 636) available with respect to the sociodemographic background variables as well as pretest and posttest scores from before and after the SPELL intervention in preschool (see *Measures*). These data show that the subgroup of children who took the national test differed substantially from those who did not (see Table 1); for instance, the former group had a greater percentage of mothers with low-mid educational levels (37%) than the latter group (31%). However, the two groups did not differ with respect to immigrant status or family income. With regard to pretest and posttest scores, the children who took the national reading test outperformed those who did not on two SPELL pretest measures (vocabulary, comprehension) and four posttest measures (deletion, letter identification, vocabulary and comprehension).

**Table 1:** Comparison of the complete sample of children in the original SPELL who did take and who did not take the reading test in second grade.

	Not taken reading test in second grade	Taken reading test in second grade	Significant difference
	(*n =* 636)	(*n =* 2700)	
Condition			
% BAU	23%	26%	
% SPELL	31%	29%	
% SPELL+Home	26%	20%	***
% SPELL+PD	20%	26%	**
Child Characteristics			
% Boys	56%	52%	*
Age (*SD*)	4.49 (*0*.*5*)	4.51 (*0*.*5*)	*
% Immigrant background	11%	10%	
Maternal education			
% Low	15%	15%	
% Low-Mid	31%	37%	**
% High-Mid	34%	34%	
% High	20%	14%	***
Paternal education			
% Low	20%	19%	
% Low-Mid	42%	48%	*
% High-Mid	21%	21%	
% High	16%	13%	*
Family income (*SD*)	631,468	618,272	
	(*525*,*412*)	(*352*,*063*)	
Preschool pretest scores			
Deletion	3.6 (*5*.*4*)	3.6 (*5*.*3*)	
Letter identification	7.0 (*3*.*6*)	6.8 (*3*.*6*)	
Vocabulary	47.0 (*18*.*2*)	49.5 (*16*.*0*)	***
Comprehension	18.2 (*4*.*6*)	18.6 (*4*.*1*)	***
Preschool posttest scores			
Deletion	6.9 (*6*.*6*)	7.5 (*6*.*5*)	**
Letter identification	8.4 (*3*.*6*)	8.6 (*3*.*3*)	*
Vocabulary	55.6 (*17*.*0*)	58.5 (*13*.*7*)	***
Comprehension	20.2 (*4*.*1*)	20.4 (*3*.*8*)	***

*Notes*. “Not taken reading test in second grade” is reference group. Percentages and mean values are calculated based on those that were not missing for the respective variables.

*<0.05

**<0.01

***<0.001.

The baseline sociodemographic characteristics of the sample analyzed in the present study are shown in Table 2 across the four study conditions. As can be seen, there are few significant differences between the children in the three treatment conditions and those in BAU. For gender, there were significantly fewer boys in SPELL (50%) and SPELL+PD (49%) than in BAU (56%). For maternal education, proportionally more mothers in the SPELL+HOME (18%) condition had low education levels than in BAU (13%). Finally, family income was significantly lower for the SPELL (596,507 DKK) and SPELL+HOME (589,720 DKK) conditions than in BAU (645,082 DKK).

**Table 2:** Baseline characteristics of children in the long-term study of SPELL.

	BAU	SPELL	SPELL+HOME	SPELL+PD
	(*n =* 701)	(*n =* 770)	(*n =* 537)	(*n =* 692)
Child Characteristics				
% Boys	56%	50%*	54%	49%*
Age (*SD*)	4.5 (*0*.*5*)	4.5 (*0*.*5*)	4.5 (*0*.*5*)	4.5 (*0*.*5*)
% Immigrant background	8%	11%+	11%+	11%+
Maternal education				
% Low	13%	17%+	18%*	12%
% Low-Mid	37%	34%	39%	38%
% High-Mid	34%	35%	30%	37%
% High	16%	13%	13%	14%
Paternal education				
% Low	16%	18%	20%+	16%
% Low-Mid	52%	46%*	50%	50%
% High-Mid	19%	22%	20%	22%
% High	13%	14%	11%	11%
Family income (*SD*)	645,082 (*387*,*434*)	596,507** (*345*,*174*)	589,720** (*355*,*988*)	618,878 (*327*,*528*)

*Notes*. BAU is reference group. Standard deviations in parentheses.

+<0.10

*<0.05

**<0.01

***<0.001.

We also examined pretest and posttest scores for children in the original four SPELL conditions based on the sample drawn for the present longitudinal analyses. As shown in Table 3, some baseline scores for the SPELL and SPELL+HOME children differed from those in the BAU condition, particularly on the vocabulary and comprehension measures. In all cases, the scores were lower for the former. Scores at posttest for three SPELL conditions also differed from the BAU group. For deletion and letter identification, the scores were higher, whereas for vocabulary and comprehension the scores were generally lower (however, for SPELL+PD the comprehension score was slightly higher). For the second grade standardized scores, children in the SPELL condition had lower language comprehension scores than those in the BAU group. Note that the comparisons presented in Table 3 do not include any covariates; thus they are purely descriptive.

**Table 3:** Pre and post test scores from the original RCT study and the national test scores for children in the long-term study of SPELL.

	BAU	SPELL	SPELL+HOME	SPELL+PD
	(*n =* 701)	(*n =* 770)	(*n =* 537)	(*n =* 692)
Preschool pre-test scores				
Deletion	3.8 (*5*.*6*)	3.3+ (*5*.*2*)	2.9** (*4*.*6*)	3.9 (*5*.*5*)
Letter identification	6.7 (*3*.*6*)	6.5 (*3*.*7*)	6.3 (*3*.*7*)	6.9 (*3*.*7*)
Vocabulary	51.3 (*15*.*3*)	48.3*** (*16*.*2*)	47.4*** (*16*.*4*)	50.6 (*15*.*9*)
Comprehension	18.8 (*4*.*2*)	18.3* (*4*.*1*)	18.3* (*4*.*4*)	18.8 (*4*.*0*)
Preschool post-test scores				
Deletion	6.6 (*6*.*6*)	7.7** (*6*.*3*)	6.8 (*6*.*4*)	8.0*** (*6*.*3*)
Letter identification	8.2 (*3*.*5*)	8.6* (*3*.*4*)	8.4 (*3*.*5*)	8.7** (*3*.*3*)
Vocabulary	59.5 (*12*.*8*)	57.8* (*14*.*3*)	56.7** (*14*.*9*)	59.1 (*13*.*3*)
Comprehension	20.4 (*3*.*7*)	20.3 (*4*.*1*)	20.1+ (*3*.*8*)	20.7 (*3*.*5*)
Reading measures in Grade 2				
Language comprehension	0.0 (*0*.*9*)	-0.1** (*1*.*1*)	0.0 (*0*.*9*)	0.1 (*0*.*9*)
Word decoding	0.0 (*1*.*0*)	-0.1+ (*1*.*0*)	0.0 (*1*.*0*)	0.0 (*1*.*0*)
Reading comprehension	0.0 (*1*.*0*)	-0.1 (*1*.*1*)	0.0 (*1*.*0*)	0.0 (*0*.*9*)

*Notes*. The table shows mean values with standard deviations in parentheses. BAU is reference group.

+<0.10

*<0.05

**<0.01

***<0.001.

### Main effects analysis

To answer the first research aim addressing the extent to which SPELL exposure during preschool may show sustained effects over time on the second grade measures of reading (language comprehension, word decoding, and reading comprehension), we estimated three mixed-effects models, one for each outcome variable, with the fixed effect covariates included at the child level. Table 4 presents the effect-size estimates for these models for the whole sample on the three second grade outcomes as compared to BAU.

**Table 4:** Effect-size estimate on the three reading scores in second grade for the three SPELL conditions.

	SPELL		SPELL+HOME		SPELL+PD	
	Coef. (SE)	*p*	Coef. (SE)	*p*	Coef. (SE)	*p*
Language comprehension	-0.07 (0.062)	0.283	0.09 (0.067)	0.179	0.10 (0.064)	0.120
Word decoding	-0.03 (0.070)	0.620	0.05 (0.074)	0.503	0.08 (0.072)	0.248
Text comprehension	-0.02 (0.069)	0.740	-0.01 (0.074)	0.899	0.10 (0.071)	0.145

*Note*. Standard errors in parentheses.

As can be seen in Table 4, there were no significant differences between the reading scores in second grade for those in any of the three SPELL conditions to those for children in the BAU condition.

### Moderation of intervention effects

To answer the second research aim concerning the moderation of long-term effects on the reading outcomes by parent and child characteristics, three sets of mixed-effects models were estimated, one for each of the three reading scores (language comprehension, word decoding and reading comprehension). Each set began by including all possible interactions of condition with parent characteristics (family income, education and immigrant status), child characteristics (age, gender), and pretests (deletion, letter identification, vocabulary, comprehension). Non-significant interaction terms were successively trimmed from the models, yielding the final models presented in Table 5. Table 5 provides the standardized coefficients (B), standard errors, and *p* values for each of the predictor variables and interaction terms. The estimated effect of each of the conditions of SPELL is the difference between the BAU condition and that SPELL condition.

**Table 5:** Moderation analyses on the three reading scores in second grade for the three SPELL conditions.

	Language comprehension		Word decoding		Text comprehension	
	Coef (SE)	*p*	Coef (SE)	*p*	Coef (SE)	*p*
SPELL	-1.21 (0.42)	0.004	-0.52 (0.21)	0.012	-0.03 (0.09)	0.765
SPELL+HOME	-0.77 (0.47)	0.097	-0.42 (0.21)	0.050	-0.13 (0.09)	0.150
SPELL+PD	-1.04 (0.43)	0.015	0.10 (0.21)	0.654	0.09 (0.09)	0.316
Age	-0.22 (0.07)	0.001	0.03 (0.07)	0.448	-0.02 (0.07)	0.584
Gender (ref. group: girl)	-0.14 (0.03)	0.000	-0.24 (0.03)	0.000	-0.28 (0.07)	0.000
Pretest scores						
Deletion	0.07 (0.02)	0.005	0.14 (0.03)	0.000	0.15 (0.02)	0.000
Letter knowledge	0.10 (0.02)	0.000	0.25 (0.02)	0.000	0.20 (0.02)	0.000
Vocabulary	0.23 (0.02)	0.000	0.09 (0.02)	0.000	0.12 (0.02)	0.000
Comprehension	-0.01 (0.04)	0.814	0.06 (0.02)	0.010	0.06 (0.02)	0.006
Family income	0.01 (0.01)	0.059	-0.00 (0.01)	0.943	0.02 (0.01)	0.001
Immigrant status (ref. group native Danes)	-0.46 (0.14)	0.001	0.05 (0.07)	0.499	0.06 (0.07)	0.440
Maternal education^a^						
Low-Mid	0.04 (0.06)	0.520	-0.18 (0.12)	0.154	-0.18 (0.12)	0.154
High-Mid	0.21 (0.07)	0.001	0.15 (0.13)	0.251	0.15 (0.13)	0.251
High	0.26 (0.08)	0.001	0.31 (0.15)	0.036	0.31 (0.15)	0.036
Paternal education^a^						
Low-Mid	0.12 (0.05)	0.034	0.07 (0.06)	0.203	0.10 (0.06)	0.072
High-Mid	0.22 (0.07)	0.001	0.24 (0.07)	0.001	0.25 (0.07)	0.000
High	0.27 (0.08)	0.001	0.24 (0.08)	0.002	0.28 (0.08)	0.001
Condition X Age						
SPELL	0.26 (0.09)	0.005				
SPELL+HOME	0.19 (0.10)	0.065				
SPELL+PD	0.24 (0.09)	0.011				
Condition X Gender						
SPELL					0.00 (0.09)	0.984
SPELL+HOME					0.23 (0.10)	0.027
SPELL+PD					0.03 (0.10)	0.761
Condition X Comprehension pretest						
SPELL	0.13 (0.05)	0.007				
SPELL+HOME	0.07 (0.05)	0.168				
SPELL+PD	0.05 (0.05)	0.353				
Condition X Immigrant background						
SPELL	0.07 (0.18)	0.700				
SPELL+HOME	0.13 (0.19)	0.488				
SPELL+PD	0.48 (0.18)	0.007				
Condition X Family income						
SPELL			0.04 (0.02)	0.013		
SPELL+HOME			0.04 (0.02)	0.019		
SPELL+PD			-0.00 (0.02)	0.938		
Condition X Maternal education^a^						
SPELL						
Low-Mid			0.35 (0.16)	0.033	0.25 (0.17)	0.123
High-Mid			0.15 (0.16)	0.350	0.13 (0.17)	0.430
High			0.19 (0.19)	0.332	0.13 (0.20)	0.490
SPELL+HOME						
Low-Mid			0.39 (0.18)	0.030	0.43 (0.18)	0.016
High-Mid			0.14 (0.18)	0.440	0.24 (0.18)	0.180
High			0.19 (0.21)	0.355	0.37 (0.21)	0.083
SPELL+PD						
Low-Mid			0.37 (0.19)	0.049	0.45 (0.19)	0.016
High-Mid			0.17 (0.19)	0.369	0.22 (0.19)	0.232
High			0.13 (0.21)	0.532	0.24 (0.21)	0.274

*Notes*.

^a.^ The interaction between education and the three outcome measures only includes children with an ethnic Danish background. Standard errors in parentheses.

Among variables in the first model, for which language comprehension served as the outcome variable, child age interacted significantly and positively with condition for children in the SPELL condition (*SD =* 0.26, *p =* 0.005, *F* = 7.73, *df =* 879896) and SPELL+PD (*SD =* 0.24, *p =* 0.011, *F =* 6.45, *df =* 430212); that is, older children in this condition benefited more than younger ones. Comprehension pretest score interacted significantly and positively with condition for children in the SPELL condition (*SD =* 0.13, *p =* 0.007, *F =* 7.40, *df =* 407476). In addition, immigrant status interacted significantly with condition for children, in that children with immigrant status benefited more than ethnic Danish children in the SPELL+PD condition (*SD =* 0.48, *p =* 0.007, *F =* 7.32, *df =* 22070).

Among variables in the second model, for which decoding served as the outcome variable, there was a significant positive interaction between income and condition for children in the SPELL condition (*SD =* 0.04, *p =* 0.013, *F =* 6.24, *df =* 754) and SPELL+HOME (*SD =* 0.04, *p =* 0.019, *F =* 5.48, *df =* 3790). For children with ethnic Danish background, there was a significant interaction between condition and maternal educational in that children in all three SPELL conditions with mothers with low-mid education benefited more than children whose mothers have the lowest educational level; SPELL (*SD =* 0.35, *p* = 0.033, *F* = 4.57, *df =* 2928.2), SPELL+HOME (*SD =* 0.39, *p =* 0.030, *F =* 4.73, *df =* 3331) and SPELL+PD (*SD* = 0.37, *p =* 0.049, *F =* 3.88, *df =* 402).

Finally, among variables in the third model, for which *text comprehension* served as the outcome variable, there was a significant interaction between gender and condition in the SPELL+HOME (*SD =* 0.23, *p =* 0.027, *F =* 4.91, *df =* 305472.4), in that boys benefited more than girls. Again, there was a significant interaction between condition and maternal educational level in SPELL+Home and SPELL+PD, in that children with mothers with low-mid education benefited more than children whose mothers have the lowest educational level; SPELL+HOME (*SD =* 0.43, *p =* 0.016, *F =* 5.78, *df =* 53576.4) and SPELL+PD (*SD =* 0.45, *p =* 0.016, *F* = 5.76, *df =* 1645.7).

None of the pretest scores–vocabulary, deletion, letter identification -significantly moderated any of the outcome variables. Neither mid-high or high maternal education level moderated the condition effect compared to low maternal education background for any of outcome variables.

## Discussion

The present study was designed to address gaps in the research literature on the effectiveness of preschool-period intervention supplements focused on language and emergent literacy skills. These include the lack of follow-up beyond one or two years after the intervention, and limited, if any, information about variability in long-term outcome associated with child and/or family factors. To address these gaps, in the present study we examined the long-term effects (3–6 years later) of the SPELL RCT conducted in Denmark which tested a language and literacy intervention supplement in a universal ECE setting delivered to an unselected sample of children [48]. In the short run, the SPELL intervention appeared to improve the language and literacy skills of both at-risk and non-at-risk children, indicating that a quality improvement in terms of providing more adequate learning opportunities in ECE settings benefitted children independent of their demographic background. Using centralized national reading assessment data, it was possible in the present study to link intervention benefits to reading outcomes in second grade and evaluate long-term impacts, both main effects and moderating variables, on later school achievement for SPELL participants. Below we summarize our main results, and highlight several major contributions of the study.

The first finding is that there was no main effect of any of the three conditions of SPELL on measures of reading at second grade. Based on analysis of the entire, heterogenous sample, including children from families with different educational background and children with non-immigrant as well as immigrant status, we can conclude that at a population level the short-term main effects faded out and did not translate into long-term advantage in acquiring reading skills. Because the preschool and second grade reading measures cannot be placed on a common underlying scale, it is not possible to determine how much of the fadeout is due to decreased rate of development after the intervention for the treatment groups and how much to catch-up for the control (BAU) condition. However, other research suggests that both phenomena are common for early intervention [30]. Note that the standardization of second grade scores reported in Table 3 is based on population norms, and the values of 0 for the BAU condition implies that the sample is broadly representative of the population with respect to these abilities.

Focusing on heterogeneous intervention effects, on the other hand, we find that a set of child and parent level factors moderated the intervention effects, depending on the outcome variable in question suggesting persistence of intervention effects for some groups of children. The most frequent interactions with condition were found for maternal education; children whose mothers were at low-mid educational level (i.e., vocational education or upper secondary education) showed a stronger effect than children whose mothers were at the lowest or higher education levels. This occurred for the outcomes of decoding (all three SPELL conditions: 0.35–0.37) and text comprehension (SPELL+HOME, 0.43, and SPELL+PD, 0.45). There was also a significant interaction with condition for immigrant status when predicting language comprehension for SPELL+PD (0.48). As immigrant children are often acquiring more than one language, their learning time is necessarily divided between Danish and a home language, so that quality of instruction may play a greater role for them than for native Danish children. Furthermore, the presence of multiple home languages increased the variability among immigrant children. The primary focus of the two additional days of intensive training received by SPELL+PD teachers was on enhancing differentiation of instruction using the specific scaffolding strategies incorporated in the program [48]. The emphasis on individual variation in SPELL+PD may have been responsible for its greater success with these children.

Children with immigrant status represented about 10% of the sample with second grade reading assessment, and children with mothers with lower education represented almost 40% of the sample. To put these findings into perspective, a recent population-representative study estimated student growth on national readings from Grade 2 to Grade 4 (national reading tests are administered every second year) to be approximately one standard deviation, or 0.5 SD per year [56]. More specifically, the yearly growth in that dataset for quintiles 1–2 (counting from the bottom) is approximately 1 standard deviation and approximately 0.5 of a standard deviation for quintiles 3–5. In other words, the effects of the intervention for children who are most often in the lower quintiles are comparable to as much as half a year of schooling (immigrant children’s language comprehension). This is a very positive finding, as these groups underperform significantly in preschool and are likely to continue to do so as they enter school.

Unexpectedly, the present findings did not reproduce the results–a negative Matthew effect–of the later analysis of the SPELL data by Dale et al. [47] which found that children with low pretest language scores (viewed as a whole) benefited most from SPELL. In fact, there were two significant results in the opposite direction in the present analysis: higher initial skills predicted higher long-term outcomes for children in SPELL, and being a boy predicted higher text comprehension for children in SPELL+HOME. This discrepancy must be due to the results for children whose low pretest scores are not associated with low maternal education or immigrant status. These children benefit more strongly than average in the short-term, but not the long-term.

In summary, although the effects of SPELL assessed on the full sample faded out completely, children of particular interest in a Danish context–children whose mothers have a lower level of education and children with immigrant background–showed significant and substantial sustained benefits of the SPELL intervention 3 to 6 years after they participated in the preschool intervention.

Bailey et al. [30] suggest that the ‘Trifecta concept´ does not include basic language and literacy skills (their Table 1) on the grounds that “they develop from natural experiences under most counterfactual conditions or are specifically targeted in universally formal or informal learning environments.” They do acknowledge that some skills may be considered as trifecta under “very adverse counterfactual conditions.” The discrepancy between the overall results of this study and the results for the two subsamples mentioned suggest that adverse counterfactual conditions might better be viewed as points on a continuum rather than a binary classification, and can vary across development dimensions, such as language, numeracy, and social development. The Danish preschool environment is certainly not ‘very adverse’ overall, but it provides limited facilitation of emergent literacy, and is therefore an intervention appropriate target. There is also research demonstrating although parents in the United States and Denmark hold similar ability and effort mindsets, they differ significantly in home learning activities, with Danish parents providing significantly less family learning activities, learning extensions, and parental time investment than US parents [57].

How can this partial and selective fadeout of SPELL be explained? Of the three factors Bailey at al. [30] mention, the requirement of a sustaining environment that it must be of such a quality that post-intervention experience can preserve initial advantage seems most relevant as a potential explanation. Variance in school quality has, for instance, been linked to sustained effects of early preschool enrollment [31]. In this study, we were not able to consider the quality of children’s primary schooling as a potential moderator of treatment effects and compare it to the role of maternal education. However, a recent follow-up study of a similar intervention supplement, LEAP [58], which found that main effects were only sustained for children whose parents had less than a college education, also found that when controlling for the quality of the sustaining school, parental background no longer predicted outcomes [59].

Other aspects of the sustaining environment that may affect persistence of intervention effects that has been suggested in the literature is misalignment between preschool and school in academic content or instructional methods. A high degree of discrepancy can disrupt the learning of children; and in the opposite direction, if the content of what is being taught in school is substantially overlapping, this may reduce the benefits of preschool learning [34]. Two results of the study may have been influenced by the degree of overlapping content. First children of mothers with the lowest levels of education did not show sustained benefit from the SPELL intervention, unlike those whose mothers were in the low-mid education category. The former group makes up approximately 15% of the sample and on average, this group of children had the lowest Danish skills prior to the SPELL intervention. At the other end of the distribution, children with mothers with high education do not show long-term effects of the intervention. This result seems less surprising as these children experience a richer learning environment at home and–based on the literature—receive more help from parents in acquiring reading in school [16]. Thus it is a plausable hypothesis that overlapping content explains why children with mothers with high education, who meet the school with skills that are beyond the level that is taught in kindergarten, do not benefit from teaching with largely repeated content. In fact, the content of kindergarten curriculum in Danish schools are similar to the content of the SPELL intervention in particular in relation to literacy (https://emu.dk/sites/default/files/2020-09/GSK_FællesMål_Børnehaveklassen.pdf). The same hypothesis may be true for children with mothers with low education but in the opposite direction. Even though SPELL *did* increase their skills in preschool, they may still meet school with too low skills to benefit from teaching.

It is, however, unclear why this group of children did not gain long-term advantage when, for instance, children with an immigrant status who had even lower (Danish) skills before the intervention do? It may be related to the lower participation of this group of children in the SPELL interventions, where both maternal education and income were associated positively with exposure [60]. But there is also considerable evidence for the association of lower maternal education with delays or difficulty in children’s language, cognitive, and social-emotional development [61] reflecting less opportunity or ability to support the development of their child, e. g., by ‘bridging’ skills from preschool and school to home. That is, low Danish scores may have different causes and consequences for children with reasonably adequate Danish input and those whose input is limited due to immigrant status.

Finally, Bailey et al. [31] list overalignment between original treatment and outcome measure and multidimensionality as related potential factors that can lead to fadeout due to lack of broader relevance of the initial outcome measure in the long term. However, the short-term effects of SPELL were found for a diverse set of emergent literacy measures which reflect current thinking concerning the preschool period foundations of literacy, and do not appear to suffer from the problem of multidimensionality. Further, the specific outcome measures were not overaligned with the intervention; for example, the outcome measure of phonological awareness was phoneme deletion, which was not used in the intervention, which focused on rhyming, alliteration, and segmentation/blending.

Several additional limitations of this study warrant mention. First, we were not able to match national reading test scores to all children in the original study, and this may have had an impact on the results. Second, because long-term effects were not evaluated for the youngest children in the original sample (the 3-year-olds), it is unknown if the present results can be generalized to children who were relatively young when receiving the SPELL intervention. Third, because of attrition relating to children who took the national test in the second grade, the sample is slightly skewed in the direction of more affluent children. Where we found interaction effects, they mainly went in the direction that less affluent children gained the most; consequently, the effects of the intervention may be slightly underestimated.

In conclusion, given that an increasing number of children are enrolled in universal ECE programs it is important to realize that large public investments in ECE programs by themselves do not guarantee a sufficiently rich learning environment for children and that more focus should be put on how to enrich the environment to ensure better short- and long-term outcomes for all children. The current study showed that when evaluated in an unselected population, enhancing the learning opportunities in ECE settings with a cost-efficient brief intervention supplement that can be readily implemented did result in some sustained improvements in reading achievement 3 to 6 years after the intervention, primarily for children exhibiting certain risk factors. Furthermore, the relative success of this intervention in the Danish context, in which pre-academic content has historically not been included in preschools [40], is encouraging with respect to its applicability in other cultural settings where it would also be highly innovative.

These positive significant long-term effects should be seen in the light of the fact that the SPELL study was carried out under real-life conditions, that is, in the context of an effectiveness rather than an efficacy trial. There was therefore significant variation in terms of intervention exposure for individual children, measured based on implementation notes reported by the teachers. Of the 40 lessons teachers were to deliver as part of the SPELL intervention, children in SPELL, SPELL+HOME, and SPELL+PD were, on average, exposed to between 58% and 70% of lessons [44]. This corresponds to an average of 12–14 hours of intervention, and yet significant long-term effects were found in some cases. A high priority for future research should be to further explore how to support implementation fidelity when implementing enhancement interventions like SPELL as this may be a cost-effective route to increased long-term benefits. Implementation of language and literacy interventions have received some recent focus [62] and one fruitful avenue to explore how to promote fidelity of interventions [63]. Based on recent work that quantified individual children’s actual learning experiences and outcomes, another promising approach to better understand the relation between persistence of intervention effects and the sustaining environment would be to get much closer estimates of actual learning opportunities. Additionally future research needs to explore the nature and degree of alignment between primary school experience and preschool intervention, along with other aspects of educational quality, as determinants of longer-term treatment effects. More attention to the environments that follow intervention will be essential to develop methods to improve persistence of early efforts over time [31].
